# Food insecurity among senior citizens in high out-migration areas: evidence from Western Nepal

**DOI:** 10.1186/s40795-020-00356-5

**Published:** 2020-06-19

**Authors:** Devendra Raj Singh, Saruna Ghimire, Eva M. Jeffers, Sunita Singh, Dhirendra Nath, Sylvia Szabo

**Affiliations:** 1 Department of Public Health, Asian College for Advance Studies, Purbanchal University, Satdobato, Lalitpur, Nepal; 2 Southeast Asian Development Actions Network (SADAN), Lalitpur, Nepal; 3 Department of Sociology and Gerontology and Scripps Gerontology Center, Miami University, Oxford, OH USA; 4 Colorado School of Public Health at the University of Northern Colorado, Greeley, CO USA; 5 Central Department of Home Science, Padma Kanya Multiple Campus, Tribhuvan University, Kathmandu, Nepal; 6 Department of Development and Sustainability, Asian Institute of Technology, Khlong Luang, Pathum Thani, Thailand

**Keywords:** Food insecurity, Senior citizens, Ageing, Out-migration, Western Nepal

## Abstract

**Background:**

Food insecurity is a critical public health challenge, particularly in low- and middle-income countries such as Nepal. The demographic transition has resulted in a growing population of senior citizens. However, the determinants of food insecurity among Nepali senior citizens remain unknown. This study aims to fill this gap by assessing food insecurity among the older populations in the far-western region, one of the poorest regions of the country. Further, we also aim to assess the potential association between adult children’s migration and the food insecurity status of the left behind older parents.

**Methods:**

A community-based cross-sectional study was conducted among 260 randomly selected senior citizens in the Kanchanpur district in far-western Nepal. The short form of the household food security scale, originally developed by the United States Department of Agriculture, was used to measure household food security. Associations were examined by logistic regression.

**Results:**

The prevalence of food insecurity in senior citizens’ households was 41.1%. Senior citizen households with their adult children’s migration (AOR = 0.47, 95% CI: 0.24–0.95) had lower odds of being food insecure whereas households with lower family income (<$100 compared to ≥ $100) had two times higher odds of being food insecure (AOR = 2.26, 95% CI: 1.08–4.76). Also, households owning a cultivable land/farm (AOR = 0.14, 95% CI: 0.05–0.40), primary source of income as service/pension (AOR = 0.26, 95% CI: 0.08–0.89) or business (AOR = 0.15, 95% CI: 0.03–0.59) and participants who received geriatric allowances (AOR = 0.05, 95% CI = 0.01–0.16) had lower odds of being food insecure.

**Conclusion:**

The prevalence of food insecurity among households with a senior citizen in Kanchanpur district was high and associated with the migration status of adult children, and household socioeconomic status. This calls for a greater policy response focused specifically on households with older adults and the integration of gerontological evidence into the existing food security and nutrition strategies.

## Background

Food insecurity is a global public health challenge and is associated with poor nutritional outcomes and health status [1–3]. Food security of individuals or households refers to “the situation when all people, at all times, have physical, social and economic access to sufficient, safe and nutritious food that meets their dietary needs and food preference for an active and healthy life” [4]. While overnutrition is increasingly a major public health issue in many developed countries [5], most of the low- and middle-income countries are still struggling to overcome hunger and undernutrition [6]. About 11% of the world population is currently affected by hunger, and the world will have to feed an additional 2.3 billion people by the year 2050 [6]. Considering this imminent challenge, the United Nations has set a goal of ending hunger, achieving food security and improving nutrition, and promoting sustainable agriculture as the second goal of its Sustainable Development Goals agenda, to be achieved by the year 2030 [7].

Nepal is one of the poorest countries, with less than US$1000 gross domestic income per capita [8]. Although agriculture is the main occupation among the Nepali population, the rapid global climate change paired with seasonal natural disasters such as flood and landslides, have severely affected the Nepalese agriculture system [9]. Consequently, food prices have increased, and access to food has become more difficult, particularly for the poorest segments of the society. Nearly five million Nepalese are directly affected by severe hunger and malnutrition [10]. Globally, Nepal ranks 72nd in the Global Hunger Index [11]. The newly promulgated constitution of Nepal of 2015 has established the provision of food security as the right of every Nepali citizen. Specifically, to address the issue of food insecurity in the country, the Government of Nepal has drafted the Food and Nutrition Security Plan of Action [12] and established a High Level Nutrition and Food Security Steering Committee to monitor the food insecurity status in the country [13]. Despite these commitments, a 2011 national survey reported about half (51%) of Nepalese households to be food insecure: 12% mildly, 23% moderately, and 16% severely food insecure [14]. Although severe food insecurity is declining (16% in 2011 to 10% in 2016), mild food insecurity is increasing (12% in 2011 to 20% in 2016) [14, 15]. Nevertheless, and irrespective of the trend, a 10% prevalence of severe food insecurity is still high enough to draw the attention of stakeholders. The country has also noted differential prevalence by rural and urban settings and ecological zones [15]. Notably, these statistics provide an overall picture of the population, and knowledge of food insecurity among senior citizens are lacking.

In Nepal, demographic transition has resulted in a burgeoning population of older adults, which comprised about 8% of the total population in the latest census of 2011 and is projected to further increase by 11% in 2030 [16, 17]. Further, the annual population growth rate of senior citizens (3.5%) is greater than the overall population growth rate (2%) of the country [16]. Nepal’s Senior Citizen Act defines people aged 60+ as senior citizens [18]. Older adults are already at increased risk of chronic health problems, which can be further exacerbated by food insecurity and poor nutritional status [19–21]. The determinants of food insecurity among older adults can be classified into five broad constructs such as intrapersonal, interpersonal, institutional, community, and sociopolitical factors [22].

Furthermore, in addition to the biological, physiological, and emotional changes accompanying the aging process, older adults in Nepal are currently facing socio-structural changes in Nepali society [23, 24]. Nepalese communities have a long-established culture and tradition of staying together in a joint/extended family, wherein multiple generations reside together, and adult sons are morally obliged to provide care and support to older parents [23, 25]. Recently, both in rural and urban areas, the conventional joint/extended family system is being replaced by nuclear families [23, 25], creating challenges regarding the care and support of older adults. Moreover, the high rates of adult children migration, both internal and external, resulted in many older parents being left alone, making them more vulnerable to negative health consequences, such as feelings of loneliness, helplessness, frustration, and increased household and social burdening [26, 27]. Contrary, the remittance received from the migrant child may uplift households’ food purchasing power and thus may increase the food security of the left behind older parents. Although no previous studies have examined food insecurity of the left behind Nepali older parents in the context of adult children’s migration, our hypothesis is based on the premise that food insecurity status is strongly correlated with poor household income or economic crisis [28]. For Nepal’s government, as well as the left-behind family members, the remittance received from foreign employment is a major source of finance [29, 30]. For families of migrant workers, remittance may help to overcome the financial crisis, and by increasing purchasing capacity, it may help to buffer food insecurity.

Studies from international settings [3, 20] suggest that older adults are more vulnerable to food insecurity. The previous studies from Nepal have focused on the general population [14, 15, 31], and to the best of our knowledge, none of the previous studies have assessed the food insecurity status among Nepali senior citizens. Moreover, food insecurity status among Nepali senior citizens in the context of adult children’s migration has not been studied before. The present study aims to fill this gap by using newly collected primary data from one of the most vulnerable regions in Nepal, far-western region. The findings of this study will contribute to future provincial and local level social security policies for senior citizens. Therefore, the objectives of this study were (1) to assess the household food insecurity status of senior citizens; and (2) identify the factors associated with households’ food insecurity among senior citizens of far western Nepal.

## Methods

### Study design and setting

A community-based cross-sectional study was conducted in the Kanchanpur district of the far-western region of Nepal from June to September 2017. The overall prevalence of food insecurity in this district in 2014, measured in terms of food poverty (measured in terms of insufficient per capita food expenditure) was 28% [32]. The district has high labor migration; almost 94% of households have at least one family member who has migrated in search of labor jobs [33]. There is a high outflow of seasonal labor migrant workers to the neighboring country of India, which borders the district in the south and west [34]. The Gulf nations and Malaysia are popular destinations for long-term labor migration for youth and adults of this district [34]. These conditions make the district an appropriate site for the study of food insecurity in the context of migration.

The study district has a total of nine local administrative units (i.e., seven urban municipalities and two rural municipalities locally known as Gaupalika). Using simple random sampling, we selected one of the nine local administrative units of Kanchanpur district. The selected Krishnapur municipality is composed of urban and semi-urban areas [35] and has 6723 households with a total population of 36,706 (17,552 males and 19,154 females); of which the population of senior citizens was 2505 (1184 male and 1321 female) [36]. Krishnapur municipality had 1861 (27.68%) households with an absent family member (absent for employment or study or business purpose), with a total of 3026 absent people (2549 male and 477 female) [36].

### Sample and inclusion criteria

The required sample size of 260 for this survey was estimated by R language-based Decision Analyst software, considering 24% prevalence of malnutrition among Nepalese senior citizens [37], 95% confidence intervals, 5% precision level, and a total population of 2505 older adults in the study area [36].

In this survey, respondents were the community-dwelling senior citizens. Criteria for eligibility included being at least 60 years old, a permanent resident of Krishnapur municipality (defined as at least 1 year of residence), and having at least one biological, step, or adopted adult child (≥18 years old). With the help of google map, surveyor started from one end of the street and selected every fifth alternate household by systematic sampling. On successive days, a different street was picked, and the same process was repeated until the desired sample size was met. One eligible senior citizen respondent was selected from each household; therefore, the number of households in the study was similar to the number of older participants. If two or more eligible participants were in one household, as is common in Nepal, the eldest was chosen. If an eligible participant was not present in the selected house, then data was sought from an eligible participant in the adjacent house. This study is the second part of the previous study that aimed to measure the association of adult children’s migration with overall well-being of the left-behind elderly parents in Nepal [38].

### Data collection, study instruments and variables

The study team collected data visiting older residents of Krishnapur municipality. Interviewers conducted face-to-face interviews in the Nepali language with individual participants to solicit information. The enumerators were public health students, and they were provided 2 days of in-person training on study objectives, data collection procedures, sample choice, tool contents, eligibility criteria, and consent process. The enumerators were familiar with the study objectives, procedure, and research ethics. The quality of the data was ensured through regular supervision of enumerators in the field, cross-checking of the collected data, and recollection of the initially missing data.

### Household food insecurity

The Six-Item Short Form of the Food Security Survey Module, originally developed and validated by the United States Department of Agriculture, was used to quantify the main outcome of this study, i.e., food insecurity of the households with senior citizen [39]. It is a continuous, linear scale variable that measures the degree of severity of food insecurity or hunger experienced in the last 12 months by a household in terms of a single numerical value [39]. The tool has been validated and used in the neighboring country, India, which provides the closest cultural settings to ours [40, 41]. The original tool was translated into the Nepali language and pretested among 26 senior citizens from the adjacent municipality before it was used. No major changes were made following the pretest. Only minor changes related to typo was corrected following pre-test. Cronbach’s alpha of the tool in this study was 0.76.

A series of six questions focused on the affordability of food in the senior citizen living households in the last 12 months. The aim was to collect the information regarding 1) concerns about food scarcity, 2) lack of resources for preferred food, 3) lack of variety of food/balanced meals, 4) eating different meals than needed/skipping meals due to lack of resources, 5) eating less meals than required due to lack of resources, and 6) not having a meal as a result of unavailability of food [39]. Each item in the food security scale was reduced to the categories of affirmative or not, as per the recommendation [39]. The sum of affirmative responses to the six questions in the module is the household’s raw score on the scale. As per the guideline, the food security status of households with raw score 0–1 was coded as food secure and two categories “low food security” (raw score 2–4) and “very low food security” (raw score 5–6) were coded as food insecure [39].

### Socio-demographic variables

The ecological conceptual framework [22] suggests five broad constructs for the determinants of food insecurity among older adults. These include intrapersonal, interpersonal, institutional, community, and sociopolitical factors [22]. Although we used this model to conceptualize our study, given the limited scope of our study, we included only the intrapersonal and sociopolitical factors that determined food insecurity among older adults. Accordingly, the independent variables included in this study are intrapersonal factors such as participants age, sex, ethnicity, family structure, migration of adult children, family monthly income, primary source of income, smoking habit, and having own cultivable land and sociopolitical factors such as participants receiving a geriatric allowance under government social protection system.

Participants’ ethnicity was classified into three major groups: Upper Caste, Janajati, and Dalit, to reflect Nepalese society’s ethnic/caste hierarchical system. Generally, Upper Caste referred to the most advantaged group, Janajatis referred to medium, and Dalit referred to the most disadvantaged group [42]. Family structure was classified as nuclear (older participant living by themselves or with a spouse), joint (older participant living with an adult child and their family), and extended family (older participant living with more than one adult child and their family in the same household). Adult children’s migration was defined as living away from the home district for the sole purpose of employment or income generation, for a period of at least 6 months, excluding the occasional visits. Socio-economic variables included self-reported family monthly income and primary income source, owning a cultivable land, and recipient of geriatric allowance. Under the social protection system, the Government of Nepal provides a monthly equivalent to US$19 old age allowance to senior citizens who are above 70 years of age [43]. However, citizens older than 60 from Dalit ethnic group and residents of the Karnali region are entitled to additional senior citizen monthly allowances [43].

### Data management and analysis

The collected data were entered into EpiData software v3.1 [44] and transferred into IBM SPSS Statistics for Window Version 21.0 (IBM Corp. Armonk, NY, USA) for statistical analyses. Based on the nature of the data, measures of central tendency and spread and frequencies were calculated. Differences in mean values and frequency distributions between food secure and insecure households were assessed using independent t-tests and Pearson’s chi-square (χ2) or Fisher’s Exact tests, respectively. Binary logistic regression model was used to analyze the presupposed association between food insecurity status and demographic and socioeconomic variables. Variables significant, at *p*-value ≤0.2, in the unadjusted models were adjusted for each other in the adjusted model.

## Results

### Demographic and socio-economic characteristics

Table 1 shows the demographic and socio-economic characteristics of households with senior citizens by food security status (Table 1). The mean (±SD) age of the participants was 68.9 ± 7.6 years. More than half of the participants were aged 60–69 years, male, from Upper Caste ethnicity, lived in a joint family, and had at least one migrant adult child. About one-third of the participants smoked. The average monthly income of the family was (median ± IQR) $80.90 ± 80.91; 65% of the households had less than $100 monthly income. Agriculture was the primary income source for more than half of the households, and more than 85% of the households owned a cultivable land. About 41% of the senior citizens received a geriatric allowance.

**Table 1:** Demographic and socioeconomic characteristics of senior citizens by household food security status

Characteristics	Total Sample ***n*** = 260	Food Security status		***p***-value
		Food Secure	Food Insecure	
		***n*** = 152 (58.5%)	***n*** = 108 (41.5%)	
	**n (%)**	**n (%)**	**n (%)**	
**Migration of adult children**				0.020*
No	127 (48.8)	65 (42.8)	62 (57.4)	
Yes	133 (51.2)	87 (57.2)	46 (42.6)	
**Age (mean ± SD)**	68.9 ± 7.6	69.4 ± 6.9	68.1 ± 8.4	0.192^**a**^
**Age category**				< 0.001*
60–69	148 (56.9)	72 (47.4)	76 (70.4)	
≥ 70 years	112 (43.1)	80 (52.6)	32 (29.6)	
**Sex**				0.739
Male	150 (57.7)	89 (58.6)	61 (56.5)	
Female	110 (42.3)	63 (41.4)	47 (43.5)	
**Ethnicity**				0.120
Upper Caste	174 (66.9)	109 (71.7)	65 (60.2)	
Janajatis	53 (20.4)	25 (16.4)	28 (25.9)	
Dalit	33 (12.7)	18 (11.8)	15 (13.9)	
**Family structure**				0.599
Nuclear	11 (4.2)	8 (5.3)	3 (2.8)	
Joint	154 (59.2)	90 (59.2)	64 (59.3)	
Extended	95 (36.5)	54 (35.5)	41 (38.0)	
**Smoking status**				< 0.001*
No	176 (67.7)	124 (81.6)	52 (48.1)	
Yes	84 (32.3)	28 (18.4)	56 (51.9)	
**Family’s monthly income (USD)**				
< 100	170 (65.4)	87 (57.2)	83 (76.9)	0.001*
≥ 100	90 (34.6)	65 (42.8)	25 (23.1)	
**Family’s monthly income (USD) (median ± IQR)**	80.90 ± 80.91	97.08 ± 85.36	64.72 ± 58.25	
**Primary income source of family**				0.012*
Agriculture	145 (55.8)	77 (50.7)	68 (63.0)	
Business	29 (11.2)	23 (15.1)	6 (5.6)	
Service /Pension/Allowance	34 (13.1)	25 (16.4)	9 (8.3)	
Wages-based labor	52 (20.0)	27 (17.8)	25 (23.1)	
**Own cultivable land**				< 0.001*
No	38 (14.6)	10 (6.6)	28 (25.9)	
Yes	222 (85.4)	142 (93.4)	80 (74.1)	
**Geriatric allowance recipient**				0.001*
No	153 (58.8)	76 (50.0)	77 (71.3)	
Yes	107 (41.2)	76 (50.0)	31 (28.7)	

a: p-value from independent t-test; others are from Chi-Square test

*: *p* < 0.05

*SD* Standard deviation, *IQR* Interquartile Range

### Food insecurity status of households with senior citizens

Approximately 41% of households with senior citizens were food insecure (Table 1). Based on the results of the adjusted binary logistic regression (Table 2), having adult migrant children (AOR = 0.47, 95% CI = 0.24–0.95), not smoking (AOR = 0.05, 95% CI = 0.21–0.13), services/pension (AOR = 0.26, 95% CI = 0.08–0.89) or business (AOR = 0.15, 95% CI = 0.03–0.59) as primary income source, owning a cultivable land (AOR = 0.14, 95% CI = 0.05–0.40) and received geriatric allowances (AOR = 0.05, 95% CI = 0.01–0.16) were significantly associated with reduced odds of being from a food insecure household. Compared to upper caste, Janajati ethnicity (AOR = 2.93, 95% CI = 1.26–6.84) had higher odds of being food insecure. Compared to a family with equal or above US$100 monthly income, a family with less than US$100 monthly income had significantly higher odds of being food insecure (AOR = 2.26, 95% CI = 1.08–4.76).

**Table 2:** Factors associated with food insecurity of households with at least one senior citizen

Characteristics	Unadjusted	Adjusted^**a**^
	OR (95%CI)	OR (95%CI)
**Age of the participants**	0.97 (0.94–1.01)*	1.06 (0.99–1.13)
**Migration of adult children**		
No	Ref	Ref
Yes	0.55 (0.33–0.91)^*^	0.47 (0.24–0.95)*
**Sex**		
Male	Ref	–
Female	1.08 (0.66–1.79)	–
**Ethnicity**		
Upper caste	Ref	Ref
Janajati	1.87 (1.01–3.49)^*^	2.93 (1.26–6.84)*
Dalits	1.39 (0.66–2.96)	0.99 (0.37–2.61)
**Family structure**		
Nuclear	Ref	–
Joint	0.93 (0.55–1.57)	–
Extended	0.49 (0.12–1.97)	–
**Smoking status**		
No	0.21 (0.12–0.36)*	0.05 (0.21–0.13)^*^
Yes	Ref	Ref
**Family’s monthly income (USD)**		
< 100	2.48 (1.43–4.30)^*^	2.26 (1.08–4.76)^*^
≥ 100	Ref	Ref
**Primary source of family income**		
Agriculture	0.38 (0.15–0.99)*	0.46 (0.19–1.10)
Business	0.28 (0.09–0.80)*	0.15 (0.03–0.59)*
Service/Pension/Allowances	0.95 (0.50–1.79)	0.26 (0.08–0.89)*
Wages-based labor	Ref	Ref
**Own a cultivable land**		
No	Ref	Ref
Yes	0.20 (0.093–0.436)^*^	0.14 (0.05–0.40)^*^
**Geriatric allowance recipient**		
No	Ref	Ref
Yes	0.40 (0.23–0.68)^*^	0.05 (0.01–0.16)*

*p < 0.05; Ref: reference category; *OR* Odds Ratio, *CI* Confidence Interval

^**a**^Adjusted for variables significant in the unadjusted model

## Discussion

This study was conducted to identify the determinants of food insecurity among households with senior citizens in far-western Nepal. The study found that 41.1% of such households were food insecure. Factors such as having an adult migrant child, not smoking, having a higher family income, having service/pension or business as a primary income source, and owning a cultivable land were associated with reduced odds of being food insecure. High prevalence of food insecurity in this study was expected given that Nepal is one of the poorest countries, with less than US$1000 gross domestic income per capita [8] and about five million Nepalese being affected by severe hunger and malnutrition [10]. Further, the far-western areas of the country are least developed compared to eastern and or central parts and most severely affected by chronic food insecurity [15, 45]. Moreover, older adults are at increased risk of food insecurity due to several factors such as financial constraints, functional limitation, disability, and social isolation [23].

In this study, adult children’s migration was associated with greater food security. We believe that the remittance from labor migration may have uplifted the households’ food purchasing power and thus increased food security. Previous studies also provide evidence that migration of adult family members for jobs and family receiving remittances back home were found to be positively associated with household food security [46, 47]. For example, a recent study by Szabo et al. on the impacts of remittance income on human-wellbeing in tropical delta regions found that households receiving remittances were significantly more likely to be food secure and had greater access to clean drinking water and sanitation [47]. Another study focusing on Bangladesh, also showed that receiving remittances was positively associated with household food security, measured by calorie intake [48].

The links between low-income status and food insecurity are well-documented [49]. Given that family’s income impacts their purchase capacity and food affordability, the association between low-income status and food insecurity observed in this study was expected and is consistent with similar studies conducted in both resource-rich and poor settings [49, 50]. In this study, several economic indicators, such as higher household income, receiving the geriatric allowance, and possession of cultivable land, were associated with lower odds of being food insecure. The finding related to owning cultivable land was expected in the Nepalese context since agriculture is mostly subsistence-based, whereby the produced food is used to meet the family needs first. This result is analogous with the study from Ethiopia, which found that small scale ownership of agriculture land has a positive effect on household food security [51].

Interestingly, smoking status was positively associated with food insecurity status. Previous studies, among a nationally representative sample of Nepalese households [52], among low-income families in Indonesia [53], and the US [54], also provide evidence of a positive association between household food insecurity and parental or family members’ smoking. This finding is concurrent with the assumption that family members’ use of tobacco products affects the household’s expenditure on food and health services in resource-poor settings [55]. However, due to the lack of total household expenditure data, this study could not explain the actual effect size of expenditure on tobacco consumption and food insecurity household food insecurity. The evidence that Nepalese households spend on an average about 5% of their annual expenditure on tobacco products [56] supports this result.

The results of this study should be viewed in light of its limitations. First, the cross-sectional design of this study does not allow for the establishment of a temporal association between food insecurity and the various demographic and socio-economic variables analyzed, and thus, no causality should be inferred. Second, the tool was developed by the United States Department of Agriculture for the assessment of food insecurity in American households and hasn’t been previously validated in the Nepali context, its validity in the Nepali context and/or among the older Nepali population may be questionable. Future studies may be needed to further assess the validity of this tool in the Nepalese context. Third, the study setting included an urban area in the far west of the country, which may limit the external validity of the study’s findings, especially in terms of similar households in rural areas and /or other parts of the country. It can be assumed that food insecurity rates might be higher in rural areas because, in general, households in rural Nepal are more likely to be poorer than urban households [57]. However, in this study, given that cultivatable land, which is more common in rural Nepal, was associated with higher food security, it is also possible that households in rural areas are likely to grow their own food and thus may have better food security status than urban. Additional studies among senior citizens in different regions of the country should be conducted for a comprehensive understanding of food insecurity; more specifically, those evaluating the hypothesized bi-directional relationship between urban-rural residency and food security status. Additionally, in a joint/extended family structure in Nepal, decision-making regarding food purchase is often done by the eldest woman in the household, often mother or mother-in-law, whereas cooking responsibility is of women-daughter in law or the eldest daughter [58]. Further, daughter-in-law or wife served the food and ate last; the oldest male member was served first. In the context of food insecurity, the serving order implies that if there wasn’t enough to eat in the household, it is younger adult women, mostly daughter-in-law or wife, who is most likely to go hungry [58]. Hence, variability in food insecurity experience may be noted even in the same household. A limitation is also felt in terms of the use of face-to-face interview for data collection as individuals may not disclose sensitive food security status due to social desirability.

## Conclusion

This study aimed at examining the determinants of food insecurity among households with senior citizens in the context of high out-migration. Our study is one of the pioneering studies to shed light on this important but understudied topic in Nepal’s context. It provided evidence that the economic migration of adult children may benefit left-behind older parents and reduce their food insecurity risks. Other key determinants of food security status amongst the household with older citizens included household socioeconomic indicators and ethnicity. These results highlight persisting inequalities in the Nepali society and, in particular, among the older population. While our results can be generalized in Western Nepal, the study findings illustrate a pressing need to conduct further research on food security status among the Nepali senior citizens – at the national level and accounting for multiple aspects of inequality. The results of this study also call for immediate action from stakeholders to support food insecure senior citizens, especially those from low-income families. As the Government of Nepal is in a preconception phase of devising national plans and policies for senior citizens, food insecurity issues should also be given priority within the agenda.

## Data Availability

All data analyzed during this study are available from corresponding author on reasonable request.

## Abbreviation

AOR: Adjusted Odds Ratio
